# Effects of multidrug-resistant bacteria and multi-antibiotic combination on intestinal microbiota in mice

**DOI:** 10.3389/fmicb.2024.1504396

**Published:** 2025-02-05

**Authors:** Jing Ma, Zheng Gong, Meiling Kang, Zhongjing Tian, Liping You, Chengshi Ding

**Affiliations:** ^1^College of Life Sciences, Zaozhuang University, Zaozhuang, China; ^2^Department of Clinical Laboratory, Zibo Central Hospital, Zibo, China

**Keywords:** multidrug-resistant *Escherichia coli*, multi-antibiotic combination, intestinal microbiota, high-throughput sequencing, antibiotics

## Abstract

Multidrug-resistant bacteria are a clinical and an epidemiological challenge. However, the effects of multidrug-resistant bacteria and multi-antibiotic combination on gut microbiota are unclear. In this study, the effects of multidrug-resistant bacteria and multi-antibiotic combination on intestinal microbiota in mice have been observed by high-throughput sequencing. Resistant *Escherichia coli* (RP4) and 0.5, 1, and 2 mg/L antibiotics (Amp, Km, and Tet multi-antibiotic combination) could decrease the number of specific operational taxonomic units from 223 in the normal saline control group to 178 in the antibiotic-resistant bacteria group and 34 in the antibiotic group, and antibiotics are the biggest influencing factor. Multidrug-resistant bacteria and multi-antibiotic combination could affect the function of intestinal microbiota, and the effect of multidrug-resistant bacteria was similar to that of multi-antibiotic combination. Small intestine is the main colonization site of antibiotic-resistant bacteria, and Proteobacteria and Bacteroidetes are the major antibiotic-resistance acquired bacteria as determined by transmission electron microscopy and agarose plate screening culture.

## 1 Introduction

Antibiotic-resistant bacteria refer to the bacteria that defend against the antibiotics. They can sustain in water, soil, air, and other environments near livestock and poultry breeding for a long time, and they transfer to other microorganisms during the processing and storage of the food, which is considered as a new food contaminant (Chee-Sanford et al., 2009; Ding et al., 2021; Marshall and Levy, 2011). At the 71st session of the United Nations General Assembly held in New York in September 2016, 193 member states unanimously adopted a declaration jointly issued by the WHO, the Food and Agriculture Organization of the United Nations (FAO), and the World Organization for Animal Health (OIE): The growing problem of antibiotic resistance should be controlled (Friedrich, 2016). The WHO released a global investigation report on antibiotic-resistant bacteria in 2014, and almost all member states reported that antibiotic-resistant bacterial infection had caused persistent infection and high mortality, which had posed a major threat to human health (World Health Organization, 2014). Antibiotic-resistant bacteria will become one of the 10 leading threats to global health in the 21st century (World Health Organization, 2023). More than 120,000 people die each year from Methicillin-resistant *Staphylococcus aureus* (Adedeji-Olulana et al., 2024). In recent years, antibiotic-resistant bacteria have evolved to resist multiple antibiotics (multidrug-resistant), and single antibiotics gradually lose their effects. Multidrug-resistant bacteria, such as NDM-1 (New Delhi metallo-β-lactamase 1) resistant bacteria, vancomycin-resistant bacteria, and tigecycline-resistant bacteria, have caused many cases of incurable diseases or even death (Diarrassouba et al., 2007; Muziasari et al., 2017). *Escherichia coli* (*E. coli*) is the most common Gram-negative bacterial pathogen, presenting both a clinical and an epidemiological challenge. In the last decade, several successful multidrug-resistant high-risk strains, such as strain *E. coli* ST131, have evolved, mainly due to the growing selective pressure of antimicrobial use (Paitan, 2018). However, the effects of antibiotic-resistant *E. coli*, especially multidrug-resistant *E. coli*, on intestinal microbiota are still unclear.

The most important factor affecting the transfer and spread of antibiotic-resistant bacteria are antibiotics, and the concentration of antibiotics in water can reach 0.917 μg/L to 0.664 mg/L (Wei, 2018). With the widespread use of artificial antibiotics in livestock and poultry production from the 1940s, antibiotic-resistant bacteria have increased significantly, and the overuse and abuse of antibiotics has accelerated this trend (Knapp et al., 2009). Amp is a *β*-lactam antibiotic, which is the earliest and most commonly used antibiotic in humans. Tet antibiotics are the most commonly used antibiotics in livestock and poultry breeding. Km is an aminoglycoside antibiotic, which is also commonly used by humans. These three kinds of antibiotics are ubiquitous in the environment, and the residues of these three kinds of antibiotics can be detected in water (Wei, 2018). The effect of a single antibiotic on gut microbiota may be more clear, but in natural environment, multiple antibiotics often coexist. So it is of more practical significance to study the effect of the combination of multiple antibiotics on gut microbiota.

Studies have shown that food is a medium for the transfer of antibiotic-resistant bacteria to humans (Liu et al., 2018). In terms of the microenvironment where antibiotics and antibiotic-resistant bacteria may exist, animal intestinal tract is an ideal place for antibiotic stress and antibiotic-resistant bacterial transfer and amplification. In this study, Illumina MiSeq PE250 second-generation sequencing platform was used to enrich and sequence the V4 region of the *16S rRNA* gene in the intestinal microbiota, observe the influence of multidrug-resistant bacteria on intestinal microbiota, analyze the correlation between environmental factors and intestinal microbiota, and predict the effect of multidrug-resistant bacteria on intestinal microbiota function.

## 2 Materials and methods

### 2.1 Materials and reagents

Kunming male mice weighing 20 ± 2 g were obtained from Huafukang Biotechnology Co., LTD. (Beijing, China). *E. coli* K12 MG1655 (RP4) (MG1655 strain) was obtained from ATCC (USA). Conjugated transfer RP4 plasmid with ampicillin resistance gene (*amp*), kanamycin resistance gene (*km*), and tetracycline resistance gene (*tet*) was used. TIANamp Stool DNA Kit Stool Genome DNA Extraction Kit was obtained from Tiangen Biochemical Technology Co., LTD. (Beijing, China), and MiniBEST Agarose Gel DNA Extraction Kit was obtained from Takara Bio (Dalian, China). Ampicillin, kanamycin, and tetracycline were obtained from Sangon (Shanghai, China). All other reagents utilized were domestic and analytically pure.

### 2.2 Instruments and equipment

A NanoDrop One Ultra-micro UV spectrophotometer and a cryo freezer ULT-7150-9 V were obtained from Thermo (Waltham, MA, United States). A T100 PCR instrument was obtained from Bio-Rad (Hercules, CA, United States). Illumina MiSeq PE250 second-generation sequencing platform (Novogene, Beijing, China) was utilized for high-throughput sequencing.

### 2.3 Grouping and feeding of animals

Mice were randomly divided into six groups: normal saline (NS) control group, antibiotic-resistant bacteria control group, antibiotic control group, antibiotic-resistant bacteria + low-dose antibiotic group, antibiotic-resistant bacteria + medium-dose antibiotic group, and antibiotic-resistant bacteria + high-dose antibiotic group. There were six male mice in each group. Each mouse was given 5 g of artificial rat food per day. The composition of the artificial rat food included corn, soybean meal, flour, wheat bran, fish meal, meat, and bone meal (water ≤ 100 g/kg, crude protein ≥ 200 g/kg, crude fat ≥ 40 g/kg, crude fiber ≤ 50 g/kg). In the NS control group, each mouse was intragastrically fed with NS 1 mL/d for 2 consecutive days and fed with distilled water. In the antibiotic-resistant bacteria control group, each mouse was intragastric for 2 days with *E. coli* K12 MG1655 (RP4) 10^8^ CFU/d and fed with distilled water. In the antibiotic control group, each mouse was intragastrically fed with NS 1 mL/d for 2 consecutive days and fed with 1 mg/L Amp, 1 mg/L Km, and 1 mg/L Tet in drinking water. In the antibiotic-resistant bacteria + low-dose antibiotic group, each mouse was fed with *E. coli* K12 MG1655 (RP4) 10^8^ CFU/d for continuous 2 d intragastric administration and 0.5 mg/L Amp, 0.5 mg/L Km, and 0.5 mg/L Tet in drinking water. In the antibiotic-resistant bacteria + medium-dose antibiotic group, each mouse was fed with *E. coli* K12 MG1655 (RP4) 10^8^ CFU/d for continuous 2 d intragastric administration and 1 mg/L Amp, 1 mg/L Km, and 1 mg/L Tet in drinking water. In the antibiotic-resistant bacteria + high-dose antibiotic group, each mouse was fed with *E. coli* K12 MG1655 (RP4) 10^8^ CFU/d for continuous 2 d intragastric administration and 2 mg/L Amp, 2 mg/L Km, and 2 mg/L Tet in drinking water. Feces were collected after continuous feeding for 16 days. This study was approved by the ethical committee of Zaozhuang University.

### 2.4 High-throughput sequencing of the *16S rRNA* gene V4 region of intestinal microbiota

The total DNA of intestinal microbiota in mice feces was extracted using a TIANamp Stool DNA Kit Stool Genome DNA Extraction Kit and identified by gel electrophoresis. For the *16S rRNA* gene V4 region amplification, the primers were AF-F: 5 ‘-GCGGAAACGactTAACTGAACC-3’ and AF-R: 5 ‘-GaAGGTCCCCCTCTTTGGTC-3’. 515F and 806R were used to amplify the *16S rRNA* gene V4 region of fecal DNA samples, and high-throughput sequencing was performed using the Illumina MiSeq PE250 second-generation sequencing platform. The original data obtained by sequencing were used as sequence tags. Interference data could be excluded, and the original data could be spliced and filtered to obtain effective tags. The effective tags of all samples were clustered. Uparse software (Uparse V7.0.1001) clustered the sequences into operational taxonomic units (OTUs) with 97% consistency and annotated the representative sequences of OTUs with species. Then, OTU clustering and species classification analysis were conducted based on effective tags. The petal pattern indicated the common and unique OTU information among groups. *t*-Test, MetaStat, LEfSe statistical treatment, quantitative insights into microbial ecology (QIIME), species difference boxplot, species complexity (evenness), and relative abundance of species in the samples were analyzed within the observation groups. The Mantel test combined with environmental factors for RDA analysis (redundancy analysis), Spearman correlation analysis, and VPA analysis (variance partitioning canonical correspondence analysis) were used to observe the correlation between diversity index and environmental factors, and obtain the environmental factors that significantly affected the intestinal microbiota (Eckburg et al., 2005; Wang et al., 2015; Wang et al., 2020).

### 2.5 Transmission electron microscopy

After 16 days of feeding, the whole intestine in the antibiotic-resistant bacteria + medium-dose antibiotic group was collected, and the outer surface of the intestine was washed with sterile NS. The small intestine was divided into the duodenum, jejunum, and ileum from front to back, and the large intestine was divided into the cecum, colon, and rectum. Different intestine segments were cut into pieces and mixed with 4 mL of sterilized NS. The sample was vortexed for 10 min to obtain bacterial suspension in NS. The centrifugation at 1000 rpm for 10 s was carried out to remove the fragments of tissues, and then the bacterial suspension was centrifuged at 5000 rpm for 10 min. The intestinal microorganisms were obtained in a precipitate form. They were fixed with 3% glutaraldehyde after washing with PBS for 12 h. The cells were washed two times with PBS and fixed in 1% OsO_4_. Then, the cells were dehydrated using the ethanol gradient method. The samples were cut into 50 nm wide slices by using an ultramicrotome and stained with 2% uranyl acetate for 1 h and lead citrate for 15 min. Finally, the images of the samples were obtained using a Hitachi JEM-1011 transmission electron microscope (Hitachi electronic, Japan) (Ding et al., 2020).

### 2.6 Functional prediction of intestinal microbiota

According to the FAPROTAX database, functional clustering heat map and relative abundance histogram predicted changes in intestinal function (Eckburg et al., 2005; Wang et al., 2015; Wang et al., 2020).

### 2.7 Validation of enteric culturable microbiota

The bacteria from different intestinal segments in the antibiotic-resistant bacteria + medium-dose antibiotic group were cultured on LB agar plates with 80 μg/mL ampicillin, 60 μg/mL kanamycin, and 50 μg/mL tetracycline. The plates were incubated at 37°C for 24 h, and the colonies were collected. High-throughput sequencing of the *16S rRNA* gene V4 region was used for the analysis of intestinal microbiota.

## 3 Results

### 3.1 OTU distribution of intestinal microbiota

In 6 groups of samples (6 in each group, a total of 36), a total of 1,522,871 high-quality sequence tags were obtained. The tag distribution of samples in each group is shown in Table 1. The length of all sequence tags was in the range of 292–302 bp. The average length of the V4 region of the bacterial *16S rRNA* gene was 292 bp, so the sequencing length of this experiment could cover the V4 region. In this experiment, OTUs were divided based on the Usearch method at 97% sequence similarity level according to the corresponding relationship between the *16S rRNA* gene sequence similarity and bacterial classification status. OTUs of each group are shown in Table 1. A total of 748 OTUs were obtained from 36 samples. The highest number of OTUs obtained per group was 566, while the lowest was 307 (Table 1). The rich OTUs indicate that the species abundance of intestinal microorganisms in the mice was very high, and the OTUs could be used for the complexity analysis of intestinal microbiota.

**Table 1:** Numbers of tags and OTUs in each group.

Group	Total tags	Taxon tags	Unclassified tags	Unique tags	OTUs
NS control	72,995	72,577	0	418	566
Resistant bacteria control	86,221	85,373	3	845	546
Antibiotics control	87,951	87,246	0	705	325
Resistant bacteria + low dose of antibiotics	86,069	85,166	0	903	491
Resistant bacteria + medium dose of antibiotics	87,351	86,960	0	391	307
Resistant bacteria + high dose of antibiotics	87,037	86,288	6	743	427

Total tags represent the total number of tags of samples in each group (effective tags, the effective data of samples in each group used for OTU clustering and other subsequent analysis); unique tags represent the number of tags with a frequency of 1 that cannot be clustered into OTUs (they cannot be used for subsequent analysis); Taxon tags represent the number of tags used to build OTUs and obtain annotation information; Unclassified tags represent the number of tags that have no common information; OTUs represent the average number of OTUs obtained from each group of samples.

### 3.2 Effect of antibiotic-resistant bacteria on the OTUs and sample complexity of intestinal microbiota

**Figure 1:**
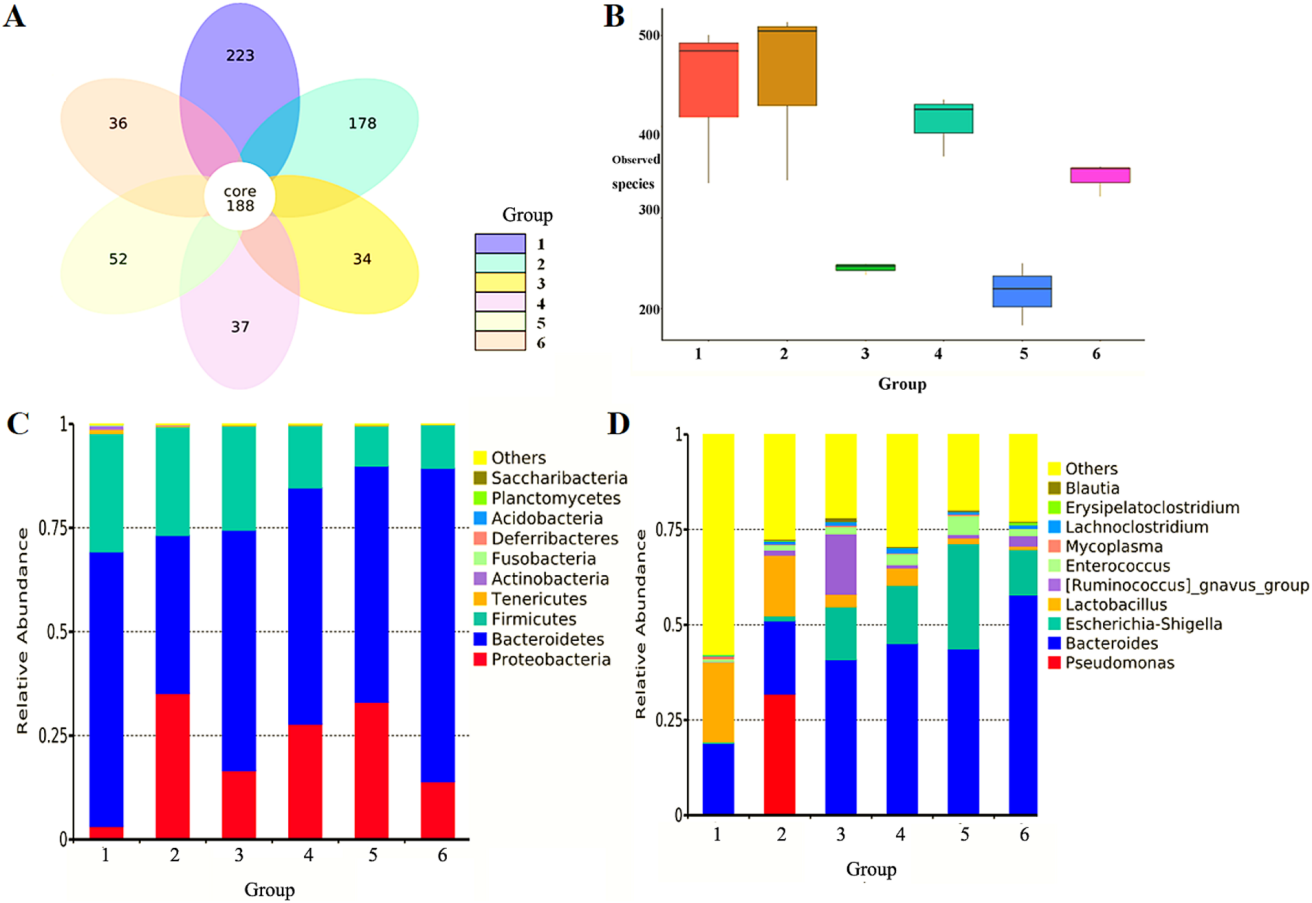
Effect of antibiotic-resistant bacteria on intestinal microbiota in mice feces. **(A)** Effect of antibiotic-resistant bacteria on OTUs of intestinal microbiota in mice. **(B)** Observed species difference boxplot of sample complexity. **(C)** Effect of antibiotic-resistant bacteria on relative abundance of dominant bacteria in the intestine of mice at the level of phyla. **(D)** Effect of antibiotic-resistant bacteria on relative abundance of dominant bacteria in the intestine of mice at the level of the genus. (1) NS control group. (2) Resistant bacteria control group. (3) Antibiotics control group. (4) Resistant bacteria + low-dose antibiotic group. (5) Resistant bacteria + medium-dose antibiotic group. (6) Resistant bacteria + high-dose antibiotic group.

Both antibiotic-resistant bacteria and antibiotics significantly reduced the abundance of intestinal microbiota in mice, and the number of specific OTUs decreased from 223 (NS control group) to 178 (antibiotic-resistant bacteria control group) and 34 (antibiotic control group). Compared with the NS control group, the combination of antibiotic-resistant bacteria and antibiotics reduced the number of bacteria-specific OTUs at all levels of dosing. The number of OTUs in the antibiotic control group (34) increased to 52 in the medium-dose group, indicating that antibiotic-resistant bacteria could resist the inhibition of antibiotics on intestinal microbiota abundance (Figure 1A).

In order to further study the effect of antibiotic-resistant bacteria on the structure of intestinal microbiota in mice, the diversity of intestinal microbiota in mice was studied from the richness and uniformity of intestinal microbiota in each group. The effects of antibiotic-resistant bacteria and antibiotics and their combined effects on the species richness and microbial diversity of intestinal microbiota were analyzed. Compared with the NS control group, antibiotic-resistant bacteria had a certain impact on the species richness of intestinal microbiota (*p* < 0.05). Antibiotics could significantly reduce the richness of intestinal microbiota (*p* < 0.01). The combined action of antibiotic-resistant bacteria and antibiotics also significantly reduced the richness of intestinal microbiota (*p* < 0.05). There was a dose-dependent relationship for antibiotics within a concentration range of 0–1 mg/L, but there was no dose-dependent relationship within a high concentration of 1–2 mg/L. Antibiotic-resistant bacteria could antagonize the decreasing effect of antibiotics on intestinal microbiota richness (Figure 1B).

### 3.3 Effect of antibiotic-resistant bacteria on the relative abundance of intestinal microbiota

The representative sequences of OTUs obtained at 97% similarity level were compared and annotated with the database in QIIME software to obtain the species annotation of each OTU. Finally, the relative abundance of each classification level in each group was counted. It was observed that Bacteroidetes, Firmicutes, and Proteobacteria were the dominant *Bacteroides* in feces of the mice in the NS control group, accounting for more than 97% of the total sequence number. The other seven phyla, Actinobacteria, Fusobacteria, Deferribacteres, Planctomycetes, Saccharibacteria, Tenericutes, and Acidobacteria, accounted for <3%. Antibiotic-resistant bacteria and antibiotics could significantly reduce the proportion of non-dominant bacteria in the seven phyla, and their relative abundance was very low (<1%). For the dominant microbiota, both antibiotic-resistant bacteria and antibiotics could reduce the proportion of Bacteroidetes and Firmicutes, and increase the proportion of Proteobacteria. Although the combined effect of antibiotic-resistant bacteria and antibiotics was consistent with the effect of the single factor on dominant bacteria, the effect was not the superposition of the two factors. Meanwhile, there was no dose-dependent manner for antibiotics (Figure 1C). The 10 genera with the largest proportion of intestinal microbiota in the NS control group were *Pseudomonas*, *Bacteroides*, *Escherichia–Shigella*, *Lactobacillus*, *Ruminococcus*, *Enterococcus*, *Mycoplasma*, *Lachnoclostridium*, *Erysipelatoclostridium*, and *Blautia*. They were the dominant bacteria in feces of mice in each group, and the proportion of the total sequence number was close to 60%. There were many other non-dominant species, accounting for more than 40%. Antibiotic-resistant bacteria could significantly reduce the proportion of non-dominant bacteria and significantly increase the proportion of dominant bacteria, especially for *Pseudomonas aeruginosa*. Antibiotics could significantly reduce the proportion of non-dominant bacteria, significantly increase the proportion of *Bacteroides*, *Escherichia coli–Shigella*, and *Ruminococcus*, and reduce the proportion of *Lactobacillus*. The combination of antibiotic-resistant bacteria and antibiotics had a similar effect on intestinal microbiota as that of antibiotics (Figure 1D).

### 3.4 Effect of environmental factors on the intestinal microbiota in mice

**Figure 2:**
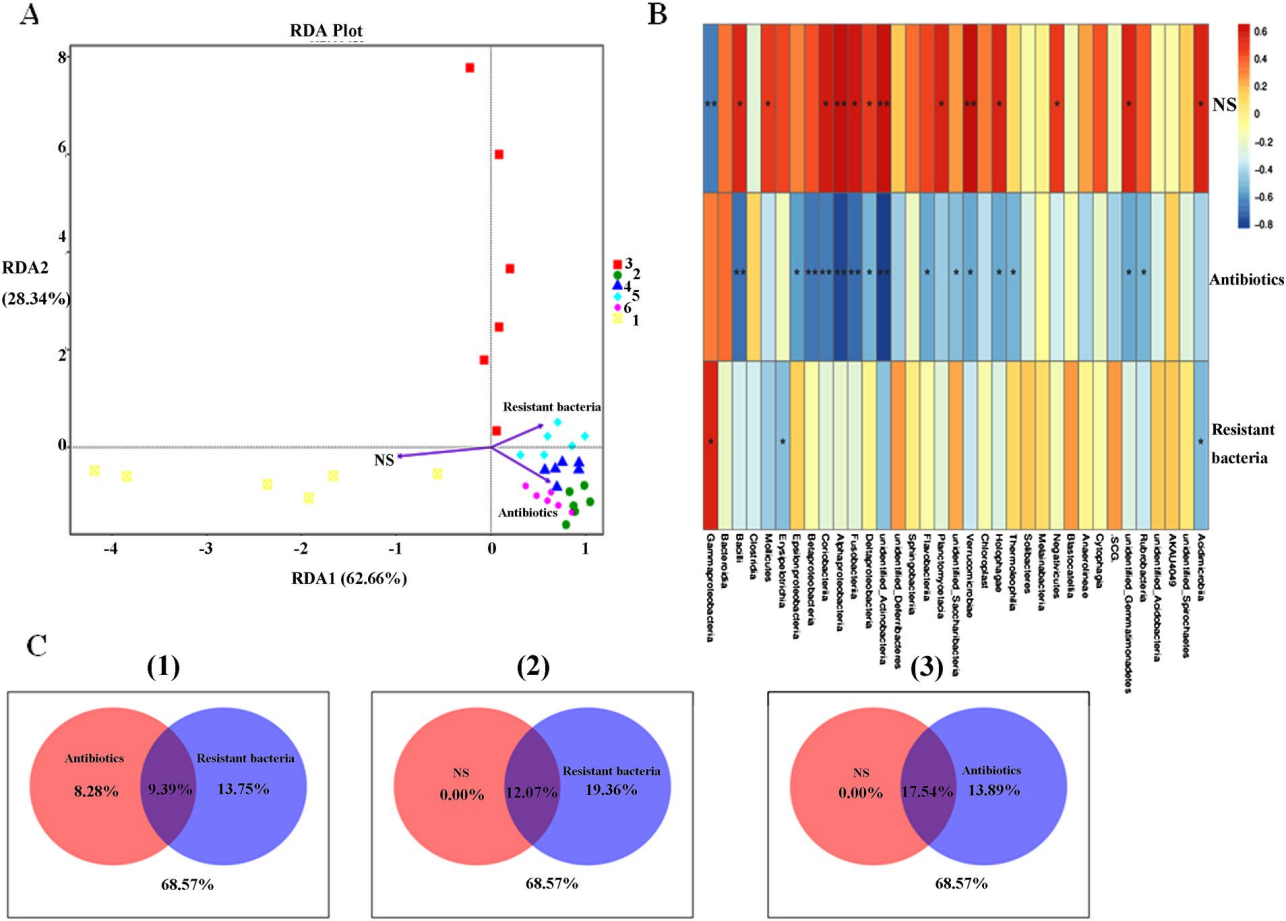
Factors of intestinal microbiota in mice. **(A)** RDA analysis. **(B)** Spearman analysis. (C) VPA analysis. (1) Antibiotics versus resistant bacteria. (2) NS versus resistant bacteria. (3) NS versus antibiotics.

Three environmental factors were designed to influence the intestinal microbiota of mice at different levels: concentration of NS (0.9 or 0 m/V), antibiotic-resistant bacteria (10^8^ CFU or 0 CFU), and antibiotic drinking water (0, 0.5, 1, and 2 mg/L) (Table 2). The samples in the NS control group were widely dispersed. The samples of antibiotic-resistant bacteria in the control group were also widely separated and were significantly distributed in different areas from those in the NS control group, indicating that antibiotic-resistant bacteria were an important environmental factor affecting the intestinal microbiota. The distribution of samples in the antibiotic control group and the resistant bacteria + high-dose antibiotic group was far from that in the NS control group, and the distance between and within the groups supplemented with antibiotics was close, indicating that antibiotics were the most important environmental driving factor affecting sample distribution (Figure 2A). The contribution of NS solution, the addition of antibiotic-resistant bacteria, and the amount of antibiotics to the intestinal microbiota was pally compared. The results showed that NS had no specific effect on intestinal microbiota (0), antibiotic-resistant bacteria had a certain specific effect on intestinal microbiota (8.28%), and antibiotics had a greater specific effect on intestinal microbiota (13.75%) (Figure 2B). Antibiotic-resistant bacteria could significantly affect the distribution of *γ*-Proteobacteria, Erysipelotrichia, and Acidimicrobiia in the intestinal microbiota of mice (*p* < 0.05). The gavage of NS can significantly affect the Bacilli, Mollicutes, Coriobacteriia, Fusobacteriia, *δ*-Proteobacteria and Planctomycetacia, Holophagae, and Acidimicrobiia (p < 0.05). It could significantly affect the distribution of Verrucomicrobiae, *α*-Proteobacteria, and γ-Proteobacteria (*p* < 0.1). Antibiotics could significantly affect the intestinal microbiota including *ε*-Proteobacteria, δ-Proteobacteria, Verrucomicrobiae, Holophagae, Thermoleophilia, and Rubrobacteria (p < 0.05). They could remarkably affect Fusobacteriia, α-Proteobacteria, Coriobacteriia, *β*-Proteobacteria, and Bacilli (*p* < 0.01). Antibiotics had a greater effect on intestinal microbiota than antibiotic-resistant bacteria and NS (Figure 2C).

**Table 2:** Effects of environmental factors on intestinal microbiota in mice.

Environmental factors	NaCl (m/V)	Antibiotics (mg/L)	Antibiotic-resistant bacteria (CFU/mL)
NS control	0.9%	0	0
Resistant bacteria control	0	0	10^8^
Antibiotic control	0	1.0	0
Resistant bacteria + low-dose antibiotic group	0	0.5	10^8^
Resistant bacteria + medium-dose antibiotic group	0	1.0	10^8^
Resistant bacteria + high-dose antibiotic group	0	2.0	10^8^

### 3.5 Characteristics of microorganisms in different segments of the mouse intestine

**Figure 3:**
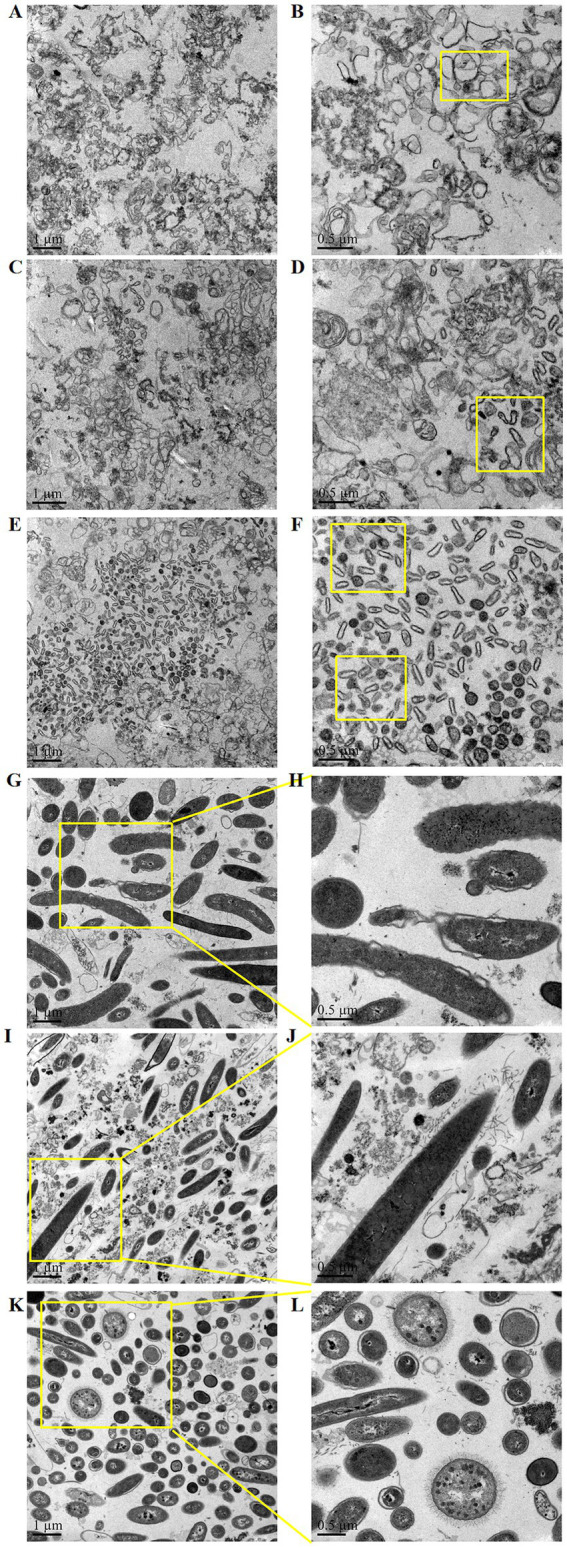
Structural characteristics of microorganisms in different segments of the mouse intestine: **(A,B)** duodenum, **(C,D)** jejunum, **(E,F)** ileum, **(G,H)** cecum, **(I,J)** colon, and **(K,L)** rectum.

In the small intestine (duodenum, jejunum, ileum), the bacteria were small in size and light in stain. The cells were clustered together and had more contact with each other (Figures 3A–F). In the large intestine (cecum, colon, rectum), the bacteria were large and deeply stained. The cell wall and membrane were thick, and the boundary was clear. The cells were evenly distributed and had little contact with each other (Figures 3G–L).

### 3.6 Functional prediction of intestinal microbiota changes in mice

**Figure 4:**
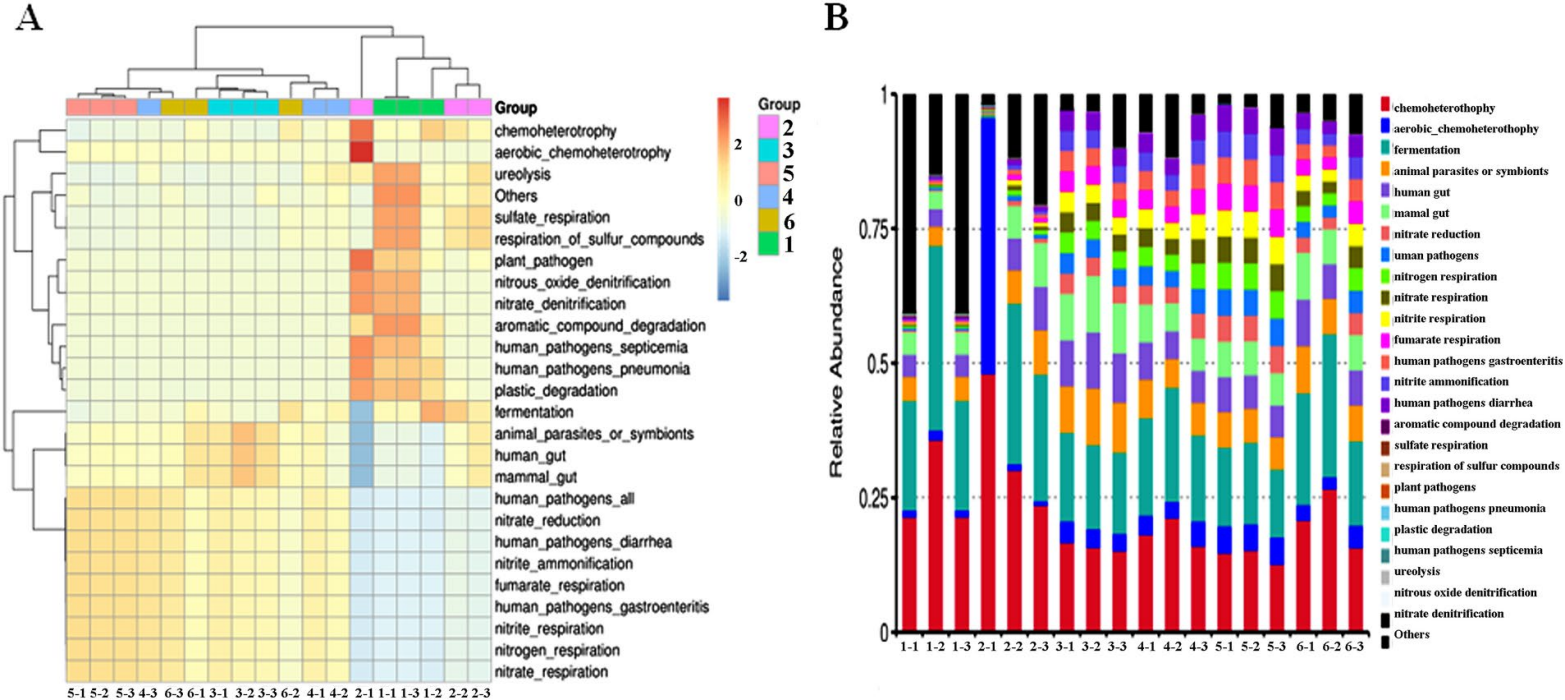
Effect of resistant bacteria and antibiotics on the function of the dominant bacteria in the intestine of mice. **(A)** Heatmap of function. **(B)** Bar plot of relative abundance of function. (1) NS control group. (2) Resistant bacteria control group. (3) Antibiotics control group. (4) Resistant bacteria + low-dose antibiotic group. (5) Resistant bacteria + medium-dose antibiotic group. (6) Resistant bacteria + high-dose antibiotic group.

Cluster analysis was conducted to explore similarities in intestinal microbiota function between groups (six groups) and within groups (three samples). The heat maps were designed to analyze changes in intestinal microbiota function between and within groups. Compared with the NS control group, antibiotic-resistant bacteria could reduce ureolysis, sulfate respiration, and respiration of sulfur compounds of intestinal microbiota. Antibiotics significantly reduced the richness of intestinal microbiota; reduced plant pathogens, nitrous oxide denitrification, nitrate denitrification, aromatic compound degradation, human pathogens septicemia, human pathogens pneumonia, plastic degradation, fermentation, animal parasites or symbionts, human gut, mammal gut-related diseases; and increased nitrate reduction, human pathogens diarrhea, nitrite ammonification, fumarate respiration, human pathogens gastroenteritis, nitrite respiration, nitrogen respiration, and nitrate respiration. The combined effect of antibiotic-resistant bacteria and antibiotics on intestinal microbiota function was similar to the effect of only antibiotic-resistant bacteria or antibiotics (Figure 4A). Compared with the NS control group, antibiotic-resistant bacteria could increase chemoheterotrophy, fermentation, animal parasites or symbionts, human gut, mammal gut, and fumarate respiration. Antibiotics could increase aerobic chemoheterotrophy, animal parasites or symbionts, human gut, mammal gut, nitrogen respiration, nitrite respiration, nitrate respiration, fumarate respiration, human pathogens diarrhea, nitrite ammonification, chemoheterotrophy, and fermentation. The results of the combined action of antibiotics and resistant bacteria on intestinal microbiota function were similar to those of antibiotics alone (Figure 4B).

### 3.7 Identification of culturable recipient bacteria in intestinal microbiota

**Figure 5:**
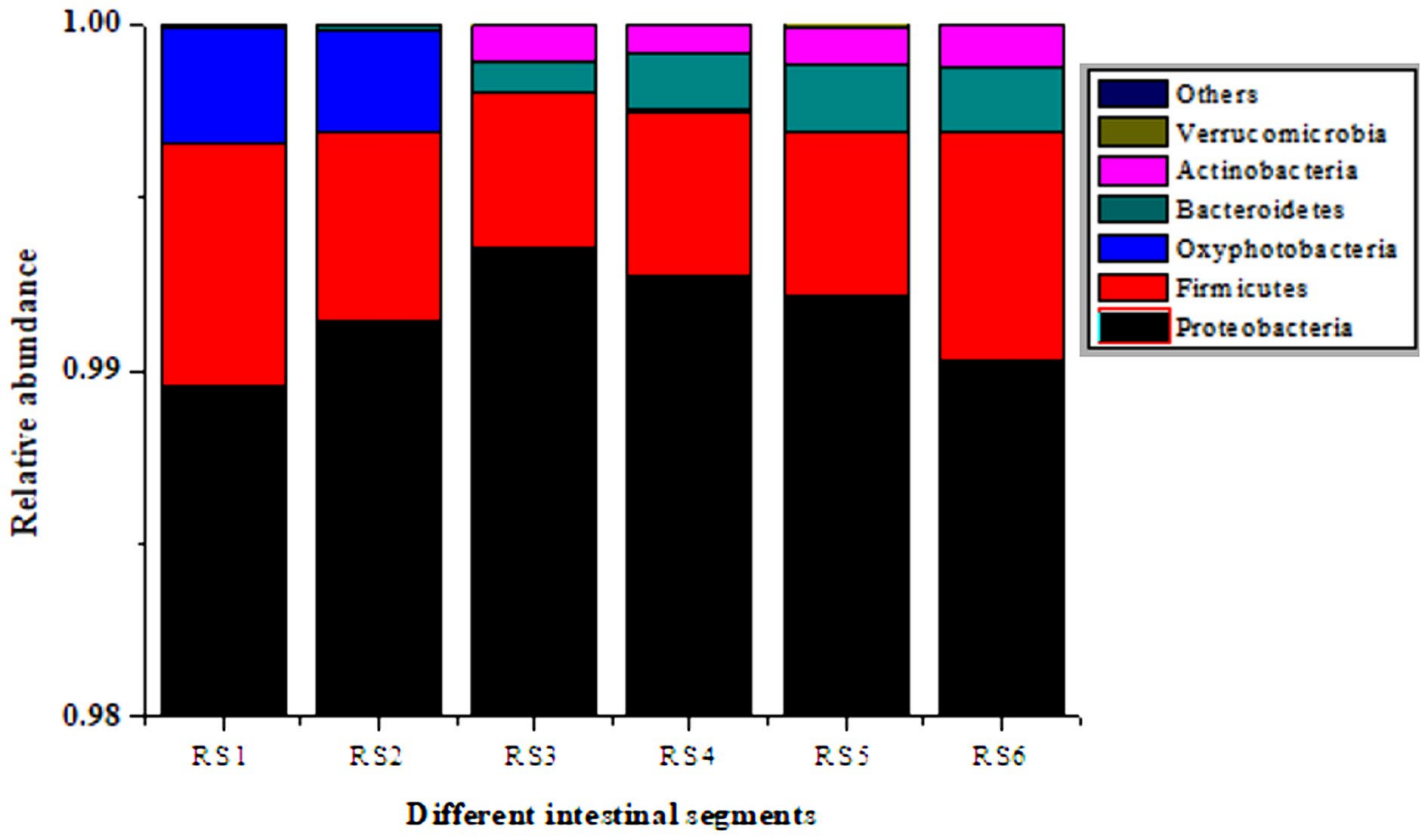
Culturable resistant bacteria in different segments of the mouse intestine. RS1, duodenum; RS2, jejunum; RS3, ileum; RS4, cecum; RS5, colon; RS6, rectum.

In the antibiotic-resistant bacteria + medium-dose antibiotic group, Proteobacteria, Firmicutes, and Bacteroidetes are the major components of the cultivable resistant bacteria in different intestinal segments. The proportion is as follows: Proteobacteria > Firmicutes > Bacteroidetes. From duodenum to rectum, Firmicutes decreased, and Proteobacteria and Bacteroidetes increased. There were fewer Lactobacillus, and more Bacillus and Enterococcus in Firmicutes. The verified results were consistent with those of intestinal microbiota analysis (Figure 5).

## 4 Discussion

Animal intestines contain a wide variety of microorganisms, and the number is huge. Intestinal microbiota has always been in a dynamic balance of interaction between itself and the animal body. Once the balance of the microbiota is broken, intestinal diseases and even systemic diseases will be caused (Van Hul et al., 2024; Ding et al., 2020; Lynch and Pedersen, 2016). Intestinal microbiota, under the action of some factors, can become antibiotic-resistant bacteria by conjugating transfer, transformation, or transduction to acquire antibiotic resistance genes (ARGs). However, complex environmental factors, such as other antibiotic-resistant bacteria (Blair et al., 2015), antibiotics (Xiao et al., 2023; Otsuka, 2020), nanomaterials (Qiu et al., 2012; Ding et al., 2016), drinking water and diet, temperature, ammonia nitrogen content, and some heavy metals (Zhang et al., 2013; Lester et al., 2006), may affect the formation and spread of antibiotic-resistant bacteria. In this study, it has been determined that multidrug-resistant bacteria and antibiotics, as new pollutants in food, will cause the colonization and transfer of antibiotic-resistant bacteria. They also will inevitably change the structure of intestinal microbiota, which may lead to the occurrence of bacterial community disorders and diseases in animals.

*Escherichia coli* (*E. coli*) is a microbe that is commonly found in food and the environment, and it can enter and grow in the intestines of animals via the food chain. Antibiotic-resistant *E. coli* is an important vector for transferring ARGs to other intestinal microorganisms. Resistant *E. coli*, as an opportunistic pathogen, is likely to affect the intestinal microbiota and lead to the risk of intestinal diseases. Although the combined effect of antibiotic-resistant bacteria and antibiotics is consistent with the effect of the single factor antibiotics on dominant bacteria, the effect is not the superposition of the two. The acquisition of multidrug-resistant bacteria can antagonize the reduction of intestinal microbiota richness caused by antibiotics. In terms of the analysis of environmental influencing factors of intestinal microbiota, antibiotics have a greater impact on the structure of intestinal microbiota than the intake of multidrug-resistant bacteria and NS. The concentration of antibiotics in water can reach 0.917–0.664 mg/L (Wei, 2018). In this study, free drinking water has been used instead of antibiotic gavage, so the concentration of antibiotics is slightly higher, which is selected as 0.5, 1, and 2 mg/L. The daily drug dosage for adults is 150–200 mg/kg, and the dosage for mice is 12.3 times that of adults (FDA, 2005). So the daily drug dosage for mice is 1800–2,500 mg/kg. Considering the daily water intake of 20–25 mL for a 20 g mouse, the concentration of antibiotics we have chosen is reasonable.

From the perspective of the bacterial community structure, previous studies have found that at the phylum level, single *β*-lactam antibiotics are characterized by the increase of Firmicutes and the decrease of Bacteroidetes and Proteobacteria under the action of antibiotics (Peng, 2014). The results of this study show that compared to the proportion of Firmicutes and Bacteroidetes, Proteobacteria increased under the combined action of a variety of antibiotics. It is speculated that under the combined action of multiple antibiotics, Proteobacteria are related to the easier access to ARGs and resistance with the help of the resistance bacteria (Ding et al., 2020; Qiu et al., 2012; Ding et al., 2016). Cefotaxime sodium, ceftriaxone sodium, levofloxacin hydrochloride, vancomycin, meropenem, azithromycin, gentamicin sulfate and ampicillin, kanamycin, and tetracycline have similar effects on intestinal microbiota at phylum level, and their differences at species level need to be further studied (Wei, 2018; Peng, 2014).

Antibiotic resistance genes (ARGs) are the genetic basis for antibiotic resistance of antibiotic-resistant bacteria, and the amplification and transfer of ARGs are the root of the spread of antibiotic-resistant bacteria (Shi et al., 2022; Ding et al., 2020). Although the number of bacteria in the small intestine is small, they are clustered in sheets. There is more contact between bacterial cells, which is convenient for conjugative transfer of ARGs. Meanwhile, the number of cells in the large intestine is large, and the contact with each other is small. The conjugative transfer of ARGs is small. Therefore, the small intestine is the main site of ARG transfer, which is consistent with the results of previous studies (Ding et al., 2020).

The balance and stability of intestinal microbiota have a significant impact on various physiological functions of the body; for example, they play an important role in nutritional digestion and absorption, immune antagonism, diarrhea, and so on (Kataoka, 2016; Sittipo et al., 2018). This study showed that the ingestion of multidrug-resistant bacteria induced animal parasitic or symbiotic diseases, mammalian and human intestinal diseases, energy metabolism diseases, and digestive and absorption diseases. Antibiotic intake may cause human diarrhea, animal parasitic or symbiotic diseases, intestinal diseases of mammals and humans, energy metabolism diseases, and digestive and absorption diseases. The results of antibiotics and resistant bacteria are similar to those of antibiotics alone. These results are based on the database of disease prediction and need to be confirmed in animal models and clinical trials.

The major antibiotic-resistant bacteria in intestinal tract were Proteobacteria, Bacteroidetes, and Firmicutes. From front to back, Firmicutes decreased, and Proteobacteria and Bacteroidetes increased. Firmicutes have thicker cell walls and are phenotypically resistant to antibiotics. Proteobacteria and Bacteroidetes may be easily resistant to antibiotics through the acquisition of ARGs, and they may be the main receptors of ARGs, which is consistent with the results of our previous studies (Ding et al., 2020).

There is less *Lactobacillus* in Firmicutes, the probiotic bacteria, from the small intestine to the large intestine. The increasing number of *Bacillus* and *Enterococcus* in Firmicutes is a conditional pathogen, and the acquisition of antibiotic resistance may make it difficult to treat infectious diseases. The intake of antibiotic-resistant bacteria will affect the stability of intestinal microbiota, destroy the balance of intestinal microecology, and affect people’s life and health. An in-depth study on the relationship between antibiotic-resistant bacteria and intestinal microbiota will provide a theoretical basis for the prevention and control of antibiotic-resistant bacteria and antibiotic contamination in food.

## Data Availability

Data is accessible within the NCBI with below accession numbers: SAMN46055412, SAMN46076568, SAMN46076570, SAMN46076585, SAMN46076598, SAMN46076613, SAMN46077354, SAMN46077356, SAMN46077360, SAMN46077435, SAMN46077442, SAMN46077444, SAMN46077445, SAMN46077466, SAMN46077568, SAMN46077592, SAMN46077612, SAMN46077661, SAMN46077678, SAMN46077720, SAMN46077800, SAMN46077808, SAMN46077890, SAMN46077913.
